# Evaluating Procedure-Linked Risk Determinants in *Trichinella* spp. Inspection under a Quality Management System in Southern Spain

**DOI:** 10.3390/ani14192802

**Published:** 2024-09-27

**Authors:** José Villegas Pérez, Francisco Javier Navas González, Salud Serrano, Fernando García Viejo, Leandro Buffoni

**Affiliations:** 1 Escuela de Doctorado, Universidad de Córdoba, 14014 Córdoba, Spain; josevillegasperez@gmail.com; 2 Departamento de Genética, Universidad de Córdoba, 14014 Córdoba, Spain; 3 Departamento de Bromatología y Tecnología de los Alimentos, Universidad de Córdoba, 14014 Córdoba, Spain; bt2sejis@uco.es; 4 Consejería de Salud y Consumo, Junta de Andalucía, 14004 Córdoba, Spain; fernando.garcia.viejo@juntadeandalucia.es; 5 Departamento de Sanidad Animal, Área de Parasitología, Universidad de Córdoba, 14014 Córdoba, Spain; h12bupel@uco.es; 6 UIC Zoonosis y Enfermedades Emergentes ENZOEM, Universidad de Córdoba, 14014 Córdoba, Spain

**Keywords:** Trichinellosis, *Trichinella*, food safety, risk level, quality management system, canonical discriminant analysis

## Abstract

**Simple Summary:**

*Trichinella* spp. is a nematode parasite that causes Trichinellosis worldwide, a foodborne zoonotic disease. To ensure high standards in food safety, a Quality Management System (QMS) was implemented in Satellite Laboratories (SLs) performing meat inspection of *Trichinella* spp. in southern Spain. The study analyzed how deviations from standard procedures may affect risk levels using Canonical Discriminant Analysis (CDA). Data were collected from audits of 18 SLs in Cordoba and Seville over six years. It was found that technical deviations, such as incorrect tests or calculations and failure to follow procedures, significantly increased risk levels. These deviations caused up to a 1.150-fold increase in risk levels, indicating their critical impact on risk determination. However, deviations related to records and documents, such as incomplete or erroneous data, did not significantly influence risk levels. The results emphasize the need to improve *Trichinella* spp. control strategies by addressing critical deviations in technique, trial information, and quality assurance to mitigate associated risks.

**Abstract:**

Trichinellosis is a major foodborne zoonotic disease responsible for 41 human cases, according to the European Union One Health Zoonoses Report. In southern Spain, a quality management system (QMS) was applied to satellite laboratories (SLs) that conduct meat inspections of *Trichinella* spp. ensuring excellence practices. This study aimed to determine how eventual deviations from standard procedures may influence risk levels using Canonical Discriminant Analysis (CDA). Data were collected during slaughterhouses and game handling establishments’ official audits in 18 SLs located in the provinces of Cordoba and Seville during a 6-year period. Technical requirement deviations regarding technique and trial information, such as performing tests or calculations incorrectly or not following technical procedures, significantly increased risk level differences. Imminent risk levels were detected if the above-mentioned deviations arose. Quality assurance compromising deviations were responsible for 1150 times risk level differences, suggesting finding such may be critical for risk determination. A lack of significant influence of records and documents compromising deviations (incomplete forms or missing-erroneous or illegible data) was found. These results strengthen *Trichinella* spp. control strategies by pinpointing crucial aspects within QMS that require improvement, particularly in addressing deviations related to technique, trial information, and quality assurance procedures to mitigate associated risks effectively.

## 1. Introduction

*Trichinella* spp. is a helminth nematode that can infest a wide variety of mammals, including humans, and it is the causative agent of the parasitic disease known as Trichinellosis. Domestic and wild pork are considered the main source of infection, although the parasite can also be found in other animals that can act as accidental hosts. In animals, the disease is usually asymptomatic, but in humans, it may cause gastrointestinal pain, fever, or muscle aches, among other symptoms. Human transmission occurs through the consumption of raw or undercooked meat containing infective L1 larvae [1]. In Spain, the wild and domestic life cycles of *Trichinella* spp. have been reported, with Iberian pigs and mainly wild boar involved as major reservoirs in each life cycle. However, it should be considered that other mammals can play an important role in transmission, such as rodents or wild animals.

Currently, this parasite continues to pose a serious threat to public health. According to the European Union (EU), in 2022, 41 human cases were documented [2]. In Spain, human cases are commonly associated with ingestion of meat from hunted wild boar. In 2022, 643 out of 212.873 (0.30%) tested wild boar in Spain resulted positive during meat inspection, whereas 2 out of 54,507,190 fattening pigs and 0 out of 812,710 breeding pigs from not controlled housing conditions resulted positive during meat inspection in Spain [2]. Furthermore, a recent study conducted in wild boar from southern Spain detected a mean seroprevalence of 6.8% over a 5-year period, ranging from 3.6% to 11.4% [3].

In 1987, the Single European Act set a deadline of 31 December 1992, for the completion of the internal market in the EU. As a result, internal border controls were abolished, and no distinction could be made between fresh meat intended for the domestic market and that intended for the intra-community market. In 1991, Directive 91/497/EEC was published, which mentioned that among the special conditions for the authorization of slaughterhouses, they must have adequately equipped facilities for *Trichinella* spp. detection. 

The importance of this zoonosis makes it considered a Notifiable Disease of an Urgent Nature, as stipulated in the National Spanish regulations and in the Regional Andalusian Autonomous Community (southern Spain) regulations. It is also considered a Public Health Alert. These considerations require that cases of Trichinellosis in humans be reported within the Public Health Alert System in Andalusia. 

Currently, the reference legislation in Europe for the investigation of *Trichinella* spp. larvae are Regulation 2015/1375 [4] and its amendments, which include the replacement of the method and by ISO Standard 18743:2015 (AECOSAN ISO 18743, 2015 [5]). According to AENOR (AENOR ISO 18743/2015, 2016 [6]), the artificial digestion/magnetic stirrer method is considered the standard method because it has been demonstrated to provide the most reliable results in validation studies [7]. 

The detection of *Trichinella* spp. larvae is an analytical determination that must provide validity and reliability in its result. The regulatory requirement for the operation of testing laboratories in accordance with the UNE/EN ISO 17025 standard [8] on General requirements for the competence of testing and calibration laboratories and their subsequent accreditation by a national accreditation body (in Spain, the National Accreditation Entity, ENAC) is addressed by Regulation (EU) 2017/625 of the European Parliament and of the Council of 15 March. This regulation deals with the designation of official laboratories, stating that competent authorities may only designate an official laboratory that operates in accordance with the UNE/EN ISO 17025 standard and is accredited accordingly by a national accreditation body. Therefore, accreditation is a requirement for *Trichinella* spp. inspection. However, laboratories whose sole activity consists of *Trichinella* spp. detection in meat may be exempt from accreditation if they are under the supervision of the competent authority. Thus, activities carried out in the internal laboratories of establishments where *Trichinella* spp. is detected, require analysis and continuous evaluation by the competent authority, and may be exempt from accreditation.

The implementation of quality assurance practices in laboratories conducting *Trichinella* spp. research is a key issue for its control. In 2019, the International Commission on Trichinellosis recommended the implementation of practices throughout the system to ensure quality [7]. However, each EU member state is responsible for implementing the minimum standards required in terms of quality assurance while considering the requirements of regulations. The evaluation of a quality management system (QMS) includes various aspects such as personnel training, diagnostic procedures, equipment and material control, and document record-keeping, among others. In addition, for the effectiveness of *Trichinella* spp. detection in meat, continuous monitoring of each critical control point is essential [9].

To promote excellent practices aimed at ensuring food safety, in 2011, the competent authorities of the Autonomous Community of Andalusia developed a QMS based on the EN ISO/IEC 17025 standard [8] and applied it to some laboratories in slaughterhouses and game meat handling establishments where tests for *Trichinella* spp. were conducted [10]. These laboratories were not accredited by ENAC but were under the supervision of the competent authority. The QMS not only allowed the incorporation of high control standards in *Trichinella* spp. analysis but also facilitated the detection of practices or habits that could be improved since this type of control had never been implemented before. Furthermore, in this study [10], we identified technical procedure issues as the primary risk factors for *Trichinella* spp. inspection in “Satellite Laboratories” (SLs). These procedures involve aspects affecting the technique or information of the trial that may influence the reliability of the result. These aspects substantiate reliable test results that are essential for public health and surveillance and may play an influence on the potential risk in terms of food quality assurance.

Therefore, the objective of this study is to conduct a retrospective analysis of the implementation of the QMS applied to slaughterhouses and game meat handling establishments from southern Spain, to determine how deviations from standard procedures influence the risk level, and to assess the potential risk these practices may entail.

## 2. Materials and Methods

### 2.1. Southern Spain’s Satellite Laboratories (SLs) Study Units and Timeframe

This study focused on evaluating the implementation of a QMS based on UNE/EN ISO 17025 [8] in 18 SLs located in southern Spain. Out of these SLs, 7 were situated in the province of Cordoba, with 4 in slaughterhouses and 3 in-game meat handling establishments. The remaining 11 SLs were in the province of Seville, comprising 9 in slaughterhouses and 2 in-game meat handling establishments. The selection of these SLs considered quantitative information, such as the number of audits conducted between 2012 and 2018, as well as qualitative factors, like the relevance of their activities and ownership (public or private). The inclusion criteria consisted of the availability of at least 4 internal audits of each SL within the specified period, except for two SLs that were audited twice during the studied period.

The SLs, which are internal laboratories within slaughterhouses and game meat handling establishments, were equipped with the necessary materials and resources to perform specific analytical determinations. Ensuring the reliability of the results was a crucial requirement to validate their validity, as mandated by EU regulations (Regulation EU 2015/1375). To guarantee this reliability, the establishments chose the option of supervision, initiating a process under the guidance of an accredited laboratory designated as the reference laboratory (Public Health Laboratory, PHL). The process involved adapting their facilities for the *Trichinella* investigation and acquiring the necessary materials and resources to align with a new approach to *Trichinella* research. The implementation of the QMS based on the UNE/EN ISO 17025 standard [8] was carried out under the supervision of the LSP, which operates under the competent authority. The successful functioning of the QMS relied on the collaboration and active participation of the economic operator (EO), the Official Veterinary Service (OVS), and the LSP.

### 2.2. Sample under Study

The study sample consisted of 3023 obtained data points gathered annually from 2012 to 2018. These observations encompassed various Types (1–6) and subtypes of deviations of the standard procedures identified in SLs during official audits [10].

### 2.3. Audits

The audits were conducted based on the criteria outlined in Regulation No. 2075/2005 of 5 December, which establishes specific standards for official controls of the presence of *Trichinella* spp. in meat. A checklist consisting of 36 questions or issues was used to gather essential information from each laboratory and evaluate their initial conditions before implementing the QMS. This checklist was created by the regional competent authority. The assessment focused on aspects such as facility requirements, technique requirements (assay), and activity data and records.

Specific documents, including procedures, instructions, and recording documents, were developed to support the QMS, aligning with the UNE/EN ISO 17025 standard [8]. During the audits, a checklist and questionnaire were employed to assess dependencies and technical requirements. An audit report was prepared to document the observed findings, categorizing them as deviations (indicating non-compliance or potential non-compliance) or improvement actions to strengthen the QMS. Corrective actions were required for both deviations and improvement actions.

### 2.4. Classification and Coding of Deviations

A classification of findings of practices that did not follow the standard procedure (named “deviations”) was previously described [10]. Briefly, the deviations were classified based on their importance (Observation—Non-conformity), aspect (Technical Requirement—Management Requirement), and affected content (execution of the technique, equipment, material, and reagents, qualification, quality assurance, records, formats, and other documents, and others). This classification aimed to group the deviations and facilitate their identification and assessment in terms of their impact on the reliability of the results, which is the primary objective of the analysis. To classify the findings, they were divided into two main groups based on whether they affected technical aspects (related to the execution of the technique) or management and documentation aspects (related to records and documents). The findings could be related to technical requirements or management requirements and varied in intensity, measured by the degree to which they affected the validity of the results, revealed non-compliance with management requirements or occurred sporadically without compromising the activities. Based on the impact, these deviations were classified as Non-conformities or Observations.

The classification of a finding as a Non-conformity (NC) or Observation (OB) depended on the auditor’s assessment of its significance or severity in compromising the outcome. Additionally, a classification based on the affected scope (management requirements/technical requirements) was performed. Within these scopes, six components were identified: Technique and/or test information, equipment, materials, reagents, qualification, quality assurance, records, forms, and other documents, as well as other components.

This categorization established six Types of findings (identified by numbers 1 to 6), with some types having subtypes (letters from a to g). Each Type and subtype encompassed specific content, providing guidance on the findings falling within their scope. The classification of deviations by Type and Subtype can be found in Table S1, which summarizes the classification system.

This classification system enables the identification of 12 distinct Non-Conformities (NC) and 12 Observations (OBS), each with their corresponding subtypes. These classifications indicate the level of compliance with the QMS and the significance of the described deviation.

Grouping the findings into specific categories based on areas and Types greatly simplifies the classification process. This approach ensures the use of a standardized criterion, promoting consistency and uniformity in the evaluation of the identified deviations. However, while this classification based on areas and Types is important, it should also consider the significance of these deviations in relation to issuing valid and reliable results. The evaluation process should account for the impact of deviations on the concepts of validity and reliability.

### 2.5. Canonical Discriminant Analysis

To identify patterns and deviations that most influence risk levels and impact the Quality Management System (QMS), Canonical Discriminant Analysis (CDA) was utilized. The CDA examined various combinations of deviation Types and subtypes to classify and group them based on their associated risk levels. The analysis considered deviations based on scope (management or technical), Type (Observation or non-conformity), and subtypes (a to g), as shown in Table S1. The clustering variable corresponded to each risk description level, detailed in Table 1.

**Figure 1:**
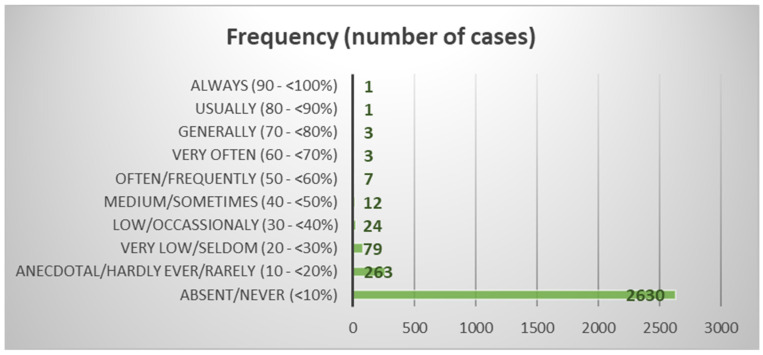
Description of deviation frequency and number of occurrences.

Figure 1 provides insights into the distribution and prevalence of deviations at different frequency levels, revealing the overall pattern and significance within the dataset. To address biases from unequal group sizes and ensure fair analysis, regularization was applied to the priors based on group sizes. This approach, using SPSS Version 26.0 for Windows (SPSS, Inc., Chicago, IL, USA), improved classification quality and prevented skewed results from imbalanced group sizes [10].

Figure 1 illustrates the frequency of deviations and the corresponding number of cases in which deviations occurred within the analyzed dataset. The deviations are categorized based on their frequency, indicating how often they occurred during the assessment process. The frequency categories are represented on the *x*-axis of the figure. The *y*-axis displays the number of cases or instances in which deviations were observed at each frequency level. The height of each bar represents the frequency count, providing an overview of the deviation occurrences.

The sample sizes in this study were robust and met established recommendations. Previous guidelines suggest a minimum of 20 observations per 4 or 5 predictors, with independent variables limited to n − 2 (where n is the sample size) to reduce distortion effects [12,13]. Our study achieved a ratio of observations to independent variables that were 4 to 5 times higher than the recommended threshold, enhancing the efficiency of the discriminant approaches. Multicollinearity was analyzed to ensure predictor independence and strong linear relationships. Forward and backward stepwise selection methods produced identical variables, but the forward selection method was chosen for its shorter runtime. Canonical discriminant analysis was conducted using SPSS version 26.0 and XSLTAT software (Addinsoft Pearson Edition 2014).

#### 2.5.1. Preliminary Testing for Multicollinearity

Multicollinearity indicates linear relationships among variables, suggesting a lack of orthogonality. Common detection methods include the variance inflation factor (VIF) and tolerance [14]. For categorical variables, VIF is calculated assuming they are continuous and dummy-coded into k − 1 dummies. This often results in k − 1 VIFs, with coefficients identified up to an unknown linear trend [14,15,16]. Collinearity among categorical variables may require splitting the dataset into reference levels, as a direct comparison of coefficients from different components is not feasible [13]. Complex collinearities among multiple factors might be better evaluated using structural equation modeling rather than traditional ANOVA [17].

#### 2.5.2. Determining the Dimension of Canonical Correlation

The maximum number of canonical correlations between two variable sets equals the number of variables in the smaller set. Typically, the first canonical correlation explains most of the relationships, but it’s important to consider all canonical correlations despite previous research often focusing only on the first [18]. A canonical correlation value of 0.30 or higher indicates about 10% of the explained variance.

#### 2.5.3. Assessing the Efficiency of Canonical Discriminant Analysis

Wilks’ lambda test is used to evaluate the significant contributions of variables to the discriminant function. As Wilks’ lambda approaches 0, the contribution of the variable to the discriminant function increases. The significance of Wilks’ lambda is tested using a χ2 test. If the significance is below 0.05, the function effectively explains the group assignment [19].

#### 2.5.4. Ensuring the Reliability of the Canonical Discriminant Analysis Model

To test the assumption of equal covariance matrices in discriminant function analysis, Pillai’s trace criterion is used, especially with unequal sample sizes [20]. Calculated as part of the Canonical Discriminant Analysis routine in XSLTAT software, a significance level of ≤0.05 indicates statistical significance. Pillai’s trace is robust against departures from multivariate normality and homogeneity of variance, with higher values indicating stronger evidence of the predictors’ significant effect on the response variable. In this study, Pillai’s trace criterion reveals potential linear differences in quality assurance traits across SL clustering groups [21].

#### 2.5.5. Interpreting Canonical Coefficients, Loadings, and Spatial Representation

In Canonical Discriminant Analysis (CDA), a preliminary principal component analysis is performed to reduce the number of variables to those most influential in differentiating SLs. CDA then determines the percentage of SLs assigned to their groups. Variables with a discriminant loading of ≥|0.40| are considered significant, while nonsignificant variables are excluded using the stepwise procedure. Variables with larger absolute coefficient values have greater discriminating ability. Data were standardized following Manly and Alberto [22]:

$$D_{ij}^{2}={(Ῡ}_{i}-{Ῡ}_{j}){COV}^{-1}{(Ῡ}_{i}-{Ῡ}_{j}),$$

where $D_{ij}^{2}$ represents the distance between population i and j; COV^−1^ is the inverse of the covariance matrix of the measured variable x; ${Ῡ}_{i}$ and ${Ῡ}_{j}$ represent the means of variable x in the ith and jth populations, respectively. The squared Mahalanobis distance matrix was converted into a Euclidean distance matrix, and a dendrogram was constructed using the UPGMA method (Rovira i Virgili University) and the Phylogeny procedure of MEGA X 10.0.5 (The Pennsylvania State University).

#### 2.5.6. Validation of Discriminant Functions through Cross Validation 

To determine the probability of an unknown SL belonging to a specific classification group [23], the hit ratio parameter is used. This parameter calculates the relative distance between the observation and the centroid of the nearest group, representing the percentage of correctly classified cases. Classification accuracy is achieved if the rate is at least 25% higher than the chance rate.

Leave-one-out cross-validation assesses the significance and validity of the discriminant functions. Press’ Q statistic supports these results by comparing the discriminating power of the cross-validated function, calculated as: $Press\;Q^{\prime }=\frac{[{n-(n^{\prime }K)]}^{2}}{n\left(K-1\right)},$ where nn is the number of observations, n′n′ is the number of correctly classified observations, and K is the number of groups. Press’ Q should be compared to the critical value of 6.63 for χ^2^ at a 0.01 significance level. A Press’ Q value exceeding 6.63 indicates that the classification is significantly better than chance.

### 2.6. Data Mining with CHAID Decision Tree: Splitting, Pruning, and Building

The CHAID decision tree, implemented using the XLSTAT software (Addinsoft Pearson Edition 2021), was used to classify and predict discrete categorized data. The tree was constructed by creating 349 internal nodes based on significant splits (*p* < 0.05) from chi-square tests during pre-pruning. Pre- and post-pruning methods were applied to avoid overfitting and include only traits that significantly contributed to the model [17]. Non-contributing nodes were pruned, and Bonferroni adjustments were used to penalize model complexity. The tree-building process involved consecutive chi-square tests to configure the tree, with branches representing test results and leaf nodes representing category levels of the target variables. Classification decisions were made at each node, starting from the root node.

### 2.7. Cross-Validation of the CHAID Decision Tree

Ten-fold cross-validation was used to validate the CHAID decision tree’s effectiveness in distinguishing group differences. The data were divided into 10 subsets, with one subset as the test set and the remaining 90% for training. This process was repeated for each subset, comparing prediction errors between the test sample and training data [16].

The complexity parameter (cp) controls tree size, halting growth if adding variables increases complexity beyond the cp value. The resubstitution rate, representing misclassified observations, decreased with tree depth, but large trees might introduce bias and overfit outliers.

Cross-validation error rates (risk) were averaged across the 10 test samples, with the tree showing the lowest cross-validation error rate selected. The optimal tree depth was identified as the shallowest tree where the cross-validation risk did not exceed the minimum risk plus one standard error [24]. This approach balanced the resubstitution and cross-validated error rates to minimize bias from overfitting.

## 3. Results

### 3.1. Multicollinearity Prevention: Preliminary Testing

As there is no presumable relationship across the levels, we decided to retain all the variables in the CDA performed in the present study to evaluate the structure of the data.

### 3.2. Canonical Discriminant Analysis

#### 3.2.1. Canonical Discriminant Analysis Model Reliability

Based on the significant Pillai’s trace criterion (Pillai’s trace criterion = 2.8488; F (Observed value) = 70.1333; F (Critical value) = 1.1197; df1 = 412; df2 = 11,676; *p*-value < 0.0001), it was determined that discriminant canonical analysis was feasible. 

**Figure 2:**
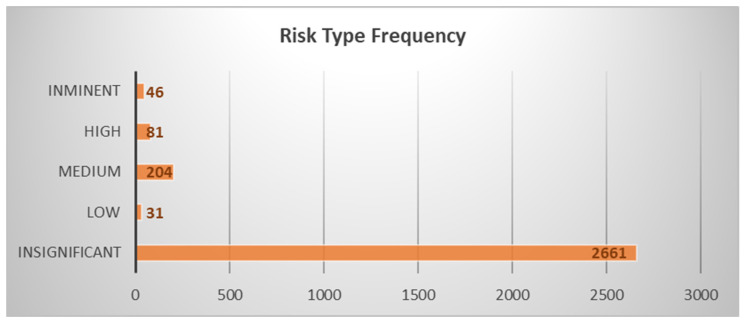
Risk description and number of occasions faced.

Table 2 shows that out of the eleven discriminant functions analyzed, seven exhibited significant discriminant abilities. Notably, the F1 function demonstrated a high discriminatory power (eigenvalue of 0.5356; Figure 2), explaining approximately 64% of the variance in combination with F2.

As reported in Table 2, the four discriminant functions designed after the analyses presented a significant discriminant ability. The discriminatory power of the F1 function was high (eigenvalue of 14.3722821), with ≈72% of the variance being explained by F1 and F2.

In Figure 2, the risk descriptions and the corresponding number of occasions in which those risks were encountered are presented. The risk descriptions are represented within parentheses and are displayed along the *x*-axis of the figure. The *y*-axis of the figure indicates the number of occasions or instances in which each specific risk was faced. The height of each bar represents the count of occurrences, providing an understanding of the frequency and significance of the risks within the dataset. Canonical relationships based on traits were utilized to visualize group differences and create an easily understandable territorial map. The selection of relevant variables was carried out using regularized forward stepwise multinomial logistic regression algorithms.

#### 3.2.2. Canonical Coefficients

Variables were ranked depending on their discriminating properties. For this, a test of equality of group means across risk level descriptions was used (Table S2). Lower values of Wilks’ lambda and greater values of F indicate a better-discriminating power, which translates into a better position in the rank. The analyses revealed either auditors’ combination or risk description significantly contribute (*p* < 0.05) to the discriminant ability of significant discriminant functions. 

##### Non-Significant (*p* > 0.05) Deviations

During audits of satellite laboratories, some deviations were found to be non-significant (*p* > 0.05) in terms of risk quantification and could be considered for exclusion. These included Non-Conformities in Management Requirement Type 5, specifically Subtype a (lacking unfilled forms or missing data that is neither erroneous nor illegible, and with the correct signatures) and Subtype b (issues with outdated data record formats or use of current primary data logging formats). Additionally, lack of findings or registration issues in Management Requirement Type 5, Subtype a, were also deemed non-significant (*p* > 0.05).

##### Type 1 Which Affects Technique and/or Trial Information

Type 1 deviations, which affect technique and/or trial information, had the greatest potential to discriminate risk levels. These deviations were mainly related to technical requirements, except for management requirement Subtype b (e.g., missing data in test reports such as temperature, sieve weight, or reagent identification), which was associated with 16 times higher risk levels. Observations and Non-Conformities in technical requirements of Subtype a (e.g., incorrect tests or use of unsupported materials) were responsible for 292- and 233 times higher risk levels, respectively. Observations and Non-Conformities in technical requirement Subtype b (e.g., missing data in test reports or inappropriate use of registry spaces) accounted for 181- and 91-times higher risk levels. The increased presence of Subtype b deviations led to risk levels that were 52 and 34 times higher. Conversely, the lack of registration of these deviations resulted in 35 times lower risk levels.

The results underscore the significance of Type 1 deviations, particularly those in Subtype a (technical requirements) or Subtype b (both technical and management requirements). These deviations compromise the reliability of results, whether by incorrect testing procedures, failing to follow technical instructions, or missing critical data. Even a single case of Type 1 deviation can significantly increase risk levels, with a single occurrence raising risk by 17 times. In contrast, Non-Conformities of Subtype a or b that are properly recorded or managed resulted in 7 and 12 times lower risk levels, respectively.

##### Type 2, Which Affects Equipment, Material, and Reagents

Within Type 2 deviations (affecting equipment, material, and reagents), the most critical findings were Observation (Management Requirement), Subtype b (absence of a Maintenance and Calibration Plan), and Observation (Technical Requirement) Subtype c (issues with equipment or consumables identification or registration), which resulted in 76 to 80 times higher risk levels compared to other deviations. Conversely, the lack of findings in Subtype e (poor condition of equipment/material), Subtype b (no Maintenance and Calibration Plan), and Subtype d (missing equipment instructions) led to 38 to 45 times lower risk levels.

Deviations affecting technical requirements, such as observations of Subtype a (no Maintenance and Calibration Plan), b (equipment/consumables issues), and d (missing equipment instructions), as well as Non-Conformities of Subtype c (missing consumable equipment/material) and management requirements of Subtype a (no Maintenance and Calibration Plan), resulted in 18 times higher risk levels. The absence of management requirement Observations in Subtype d (equipment instructions present) led to almost 14 times lower risk levels.

The presence of multiple management requirement deviations of Subtype b (equipment/consumables issues) and d (missing equipment instructions) caused about 10 times higher risk levels, while the absence of technical requirement Observations of Subtype c (missing consumable equipment/material) led to 10 times lower risk levels. Additionally, the presence of technical Non-Conformities of subtypes a (no Maintenance and Calibration Plan) and d (missing equipment instructions), or Observations of Subtypes e (poor condition of equipment/material) and f (lack of reagents), resulted in approximately 7 times higher risk levels. The absence of management requirement observations in Subtype b (equipment/consumables issues) was associated with 5 times lower risk levels, while increased presence of management Observations of Subtype b or Observations of Subtype c and Non-Conformities of Subtype d contributed to about 3 times higher risk levels.

##### Type 3 Which Affects Qualification

The lack of registration of either Non-Conformities or Observations of Type 3, for which there is no subtype, means it compromises the evidence of the qualification or training of the person(s) involved in the performance of the technique and/or the interpretation of the result, implied 38 and 33 increased risk levels if these concerned technic requirements while when Observations concerned management requirements risk levels increased by 5 times.

##### Type 4 Which Affects Quality Assurance

The presence of technical requirement deviations or Non-Conformities of Subtype c (concerned with calibrations, verifications, maintenance, or calibration labels) and management requirement Observations of Subtype c (related to similar aspects) and Subtype f (record of deviations) resulted in risk levels 103 to 170 times higher. Conversely, the lack of management requirement Observations of Subtype a (internal or external quality controls) and Subtype d (quality certificates or technical sheets) and technical requirement Non-Conformities of Subtype a led to 63 to 93 times lower risk levels. Additionally, missing Subtype e (ISO certificates of suppliers) technical requirement Observations resulted in 37 times lower risk levels.

Technical requirement Observations of Subtype a (internal or external quality controls) increased risk levels 19 times, while Subtype a Non-Conformities increased them 9 times. Subtype b (Corrective Action Plan or management of deviations) Observations raised risk levels 9 times, and Subtype a Non-Conformities increased risk levels 12 times. The presence of Subtype b management requirements resulted in a 59-fold increase in risk levels. The increased presence of Subtype c technical requirement Non-Conformities and Observations (concerned with calibrations and verifications) led to 59 and 19 times higher risk levels, respectively. Observations of Subtype e (ISO certificates) and Non-Conformities of Subtype d (quality certificates) or g (reagent conservation) raised risk levels 48, 28, and 19 times, respectively. The increased presence of technical or management requirements of Subtypes c (calibrations and verifications), d (quality certificates), or f (record of deviations) was associated with 9 times higher risk levels.

##### Type 5 Which Affects Records, Formats, and Other Documents

The absence of Observations of management requirements of Subtype c (technical instructions), d (technical procedures or regulations), and f (document control) resulted in risk levels 121 to 170 times lower. Similarly, missing technical requirement Observations of Subtype c and f led to around 64 times lower risk levels, while missing Observations of Subtype b and d resulted in 55 to 60 times lower risks. Lack of findings for Subtype a (complete and correct forms) and e (no deletions or unvalidated corrections) implied 10 and 38 times lower risks, respectively.

The absence of Subtype b management requirement Observations (data record formats) decreased risk levels by 42 times. In contrast, the presence of Subtype a management requirement Observations (unfilled forms) increased risk levels 7 to 80 times, with no significant increase for multiple occurrences. Subtype a management requirement Non-Conformities raised risk levels around 7 times, and further findings did not significantly alter risk levels. Similarly, Subtype b management requirement Observations and Non-Conformities (data record formats) were associated with 3 to 40 times higher risks, while Subtype b technical requirement Non-Conformities led to 3 to 4 times higher risks. Subtype c technical requirement Observations and Subtype e management requirement Observations increased risk levels by around 7 times.

#### 3.2.3. Standardized Loading Interpretation and Spatial Representation

**Figure 3:**
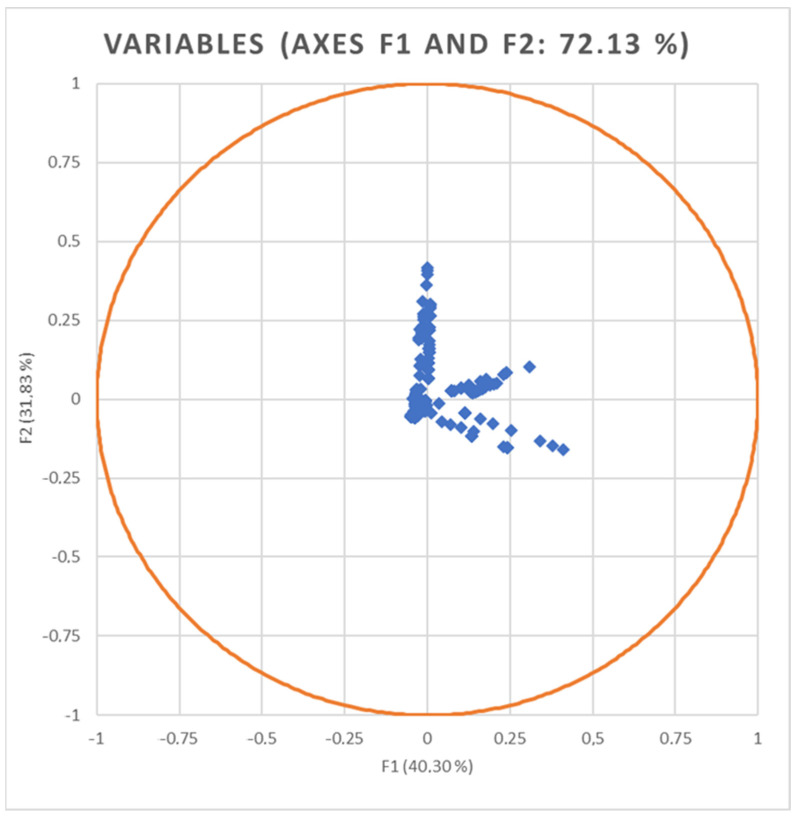
Standardized canonical discriminant function loadings shows the loading of each analyzed deviation.

Standardized discriminant loadings measure the relative weight of deviation combinations in the discriminant functions (Figure 3).

As suggested in Table S2, only the two most relevant functions were used to build a standardized discriminant coefficient biplot, capturing the highest fraction of variance (Figure 1). In this regard, those variables whose vector extends further apart from the origin most relevantly contributed to the first (F1) and second (F2) discriminant functions.

**Figure 4:**
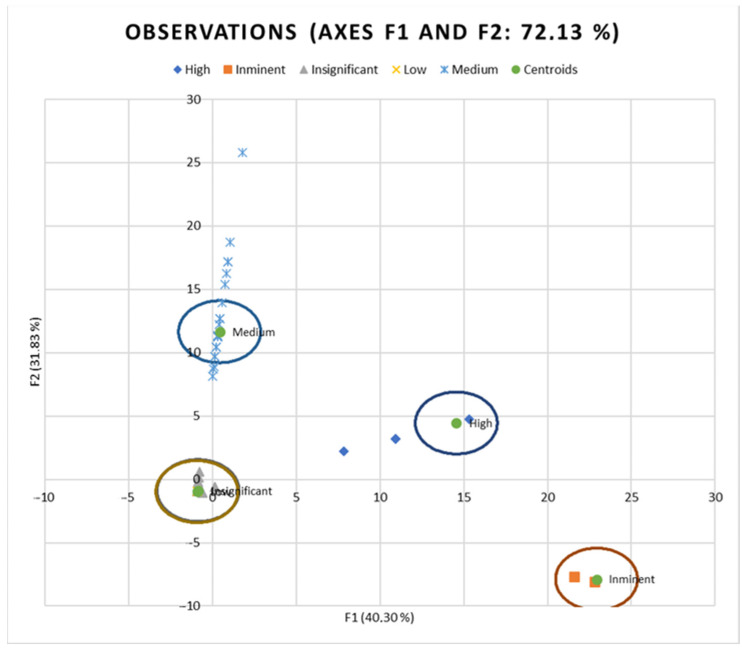
Territorial map with deviations sorted across risk levels shows detected deviations grouped by risk levels.

Table 3 and Figure 4 suggest clear differentiation among the risk levels considered in the analyses. The relative position of centroids was determined through the substitution of the mean value for observations in each term of the first two discriminant functions (F1 and F2). The larger the distance between centroids, the better the predictive power of the canonical discriminant function in classifying Observations.

**Figure 5:**
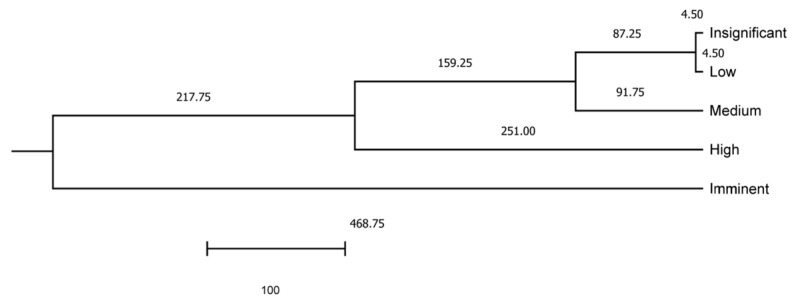
Representation of Mahalanobis distances across risk levels shows Mahalanobis distances across risk levels.

Additionally, to evaluate the proximity between risk levels, Mahalanobis distances were represented (Figure 5). 

Two main clusters are formed, the first represented by Imminent risks, which was the most distant level from the rest (Mahalanobis distance of 468.75) when deviation combinations are considered, and a second cluster comprising the rest of the risk levels (217.75). Two subclusters are formed within the second cluster, the first at 251.00, comprising High-risk levels, and the second one comprising Medium, Low, and Insignificant risk levels at 159.25. It is within the second subcluster where two subsubclusters are formed. The first subsubcluster comprises medium risk levels at 91.75, and a second subsubcluster (87.25) accounts for two additional branches at 4.50, comprising Low and Insignificant risk levels, respectively.

**Figure 6:**
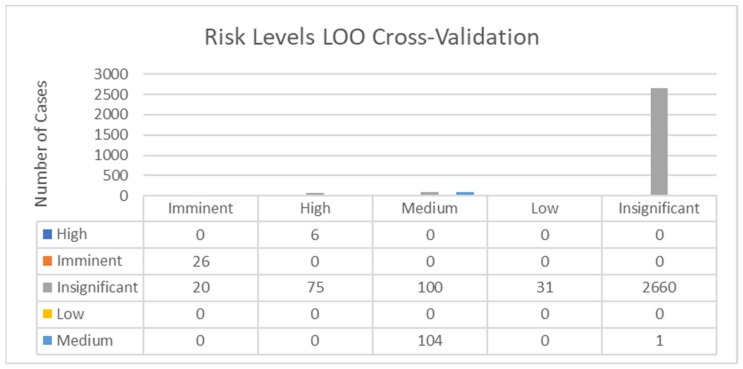
Cross-validation results within risk levels. Each group of columns represents the number of correctly and incorrectly classified deviations across risk levels.

Figure 6 reports the results obtained in the classification and leave-one-out cross-validation. A Press’ Q value of 534.804 (N = 3023; n = 2796; K = 5) was obtained when risk levels were considered the clustering criterion. Therefore, it can be considered that predictions were significantly better than chance at 95% [25].

All the cases which were determined as High or Imminent were correctly classified. Despite most of the cases classified as Insignificant being correctly classified (92.17%), 20 cases considered as Imminent were indeed Insignificant; 75 cases labeled as High were indeed Insignificant, 100 cases classified as Medium were indeed Insignificant and 31 cases classified as Low were indeed Insignificant, respectively. None of the cases classified as Low acted as low-risk cases. All the cases classified as Medium were correctly classified except for 1, which was an Insignificant risk-level case.

## 4. Discussion

Meat inspection for *Trichinella* spp. is based on direct detection of the parasite (L1) in muscle tissue and performed in specialized laboratories equipped and authorized for this purpose by the competent authority and according to EU regulations. The procedure is complex and involves various critical control points. Hence, assurance quality practices are fostered to meet the required standards [9]. In this context, any failure during the process is likely to increase the risk. Depending on the severity and the frequency of occurrence, some events may cause different levels of risk.

To analyze the potential risk implied by findings resulting from the audits carried out in the SLs conducting meat inspection of *Trichinella* spp. we have analyzed different Types of variables that did not meet the required standard procedures as previously described [10]. These variables were defined as “deviations” and addressed all aspects performed in the SLs during *Trichinella* diagnosis within the scope of a QMS, including management and technical requirements.

Overall, during official audits, different types (and subtypes) of deviations were recorded at the SLs, some of them not statistically significant and hence not considered risk determinants of any kind. By contrast, some findings were considered statistically significant, eliciting different levels of risk, ranging from imminent to low, according to Villegas-Pérez et al. [10].

Firstly, the CDA showed that some deviations did significantly affect the risk level. For instance, when procedures regarding Type 1 deviations were identified during audits, a significant increase in the risk level was observed. These proceedings involve the technique and trial information. When all procedures included within Type 1 are identified during audits, the risk level increases 1019 times (as shown in Table S1). Furthermore, in the event that one deviation is identified as a technical requirement (either OBS or NC) within Type 1 Subtype a, the risk level increases over 500 times. These findings indicate that this category of procedures is of utmost importance for risk analysis, given that these procedures are responsible for over 500 times the differences across risk levels (Table 2).

Procedures affecting equipment, materials, and reagents (Type 2) also showed a significant impact on the risk level. Indeed, a significant increase in 500 times the risk level was observed when Type-2 procedures were identified, suggesting these deviations play a significant role in risk assessment.

Qualification and training of the person involved in the performance of the technique and/or the interpretation of the result is a major aspect of *Trichinella* spp. inspection. As indicated by the International Commission on Trichinellosis [7], any personnel conducting the mandatory testing methods outlined in EU 2015/1375 (European Commission. Commission Implementing Regulation 2015/1375) should be previously trained to meet the standard requirements. In our study, we observed that deviation within this scope (Type 3) did slightly increase 75 times the potential risk, although at a lower level compared to procedures included in Type 1–2. Usually, veterinarians are in charge of carrying out the mandatory testing procedure at internal laboratories. In the context of quality assurance practices included in a QMS, a previous training activity of these highly qualified professionals should enable them to meet the minimum required standards. Furthermore, the performance of laboratories depends on personnel expertise [10].

In terms of deviations affecting the quality assurance (Type 4), we observed a significant influence on the risk level, though depending on the specific activity (subtype) affected, that is, all subtypes of deviations affecting quality assurance significantly increased the risk but at different levels. When considered altogether, an increase of 1150 times in the risk level was observed, which indicates these procedures as being one of the most influencing factors within a QMS. Additionally, when individual subtype deviations were analyzed, a significant difference was observed, suggesting all regarded procedures play a significant role in the influence of the risk level, regardless of the affected activity.

With respect to deviations affecting records, formats, and other documents (Type 5, Subtypes a and b, [10]), no significant differences were detected, indicating a lack of influence on the risk level. This result suggests that deviations included within the above-mentioned scope are less relevant than other deviation types in terms of influence on the risk level. Consequently, during audits and within the QMS, we suggest strengthening and focusing on those aspects that show a higher influence on the level of risk and simplifying the procedures that involve records and documentation. It is worthwhile noting that Subtypes a and b regard the internal records and documents of each SL and are different from force-mandatory documents in terms of official communication to the competent authority when a positive diagnosis occurs. On the contrary, deviations considered in force-mandatory procedures within Type 5 did increase up to 1178 times the risk level, which highlights the major influence of these aspects.

When comparing the risk level among the different types of deviations, in force procedures, as well as deviations affecting the technique and trial information, the equipment, material and reagents, and the quality assurance resulted in a significant increase of the risk level. We suggest these results might be used to establish a risk classification of establishments performing *Trichinella* spp. inspection. Indeed, the competent authority of southern Spain has developed a procedure that can be used to classify food establishments based on risk assessment and, consequently, to determine the frequency of audits as a tool to improve quality assurance (Regulation 18/2012, Procedure for the classification of food establishments based on risk in Andalusia, Andalusian Autonomous Community). Furthermore, García Gómez [26] claims that decisions regarding food safety are to be based on risk analysis as consumer behavior regarding food is highly conditioned by risk perception, evaluation, and management. Similarly, Pozio [27] states that the competent authority should apply a surveillance system for *Trichinella* spp. control based on risk assessment.

Establishments conducting *Trichinella* spp. detection plays a pivotal role in terms of food quality and safety, and all stakeholders should be encouraged to evaluate their potential level of risk to ensure food safety. To date, unfortunately, there are no studies addressing the influence of the lack of compliance with standard procedures occurring in laboratories performing *Trichinella* spp. inspection, in the context of official audits, on the risk level. However, there are some reports approaching risk assessment of laboratories in public health: Graça et al. [28] developed a method to study the risk level of analytical laboratories (microbiological laboratory and water analytical laboratory) based on in situ data from testing procedures. Furthermore, a laboratory quality control guideline has been developed based on risk management [28]. It should also be considered that risk level may also be influenced at a farm level. Although our study did not focus on this aspect, a thorough, comprehensive analysis of procedures conducted at all stages of the food industry might provide a further understanding of *Trichinella* spp. risk level.

On the other hand, Bai et al. [29] recently reported that creating an indicator system is the first step to assessing risk. These researchers created a method based on a mathematical model to evaluate the quality risk level in the food supply chain. This quality risk indicator system for the food supply chain covers five evaluation objectives and 55 quality risk evaluation indicators and was built to provide a basis for evaluating the food quality risk level. Moreover, using different indicators may provide inputs to accurately assess food safety risk levels, such as those recently reported by Salvo et al. [30], who used toxic inorganic pollutants in food to evaluate risk. Likewise, Etter et al. [31] highlighted the importance of risk assessment as a decision tool for the management of zoonotic disease.

It is worth acknowledging that our study focused on one link of the whole food supply chain. Further studies considering a comprehensive and systematic approach to the risk evaluation of the food supply chain that includes all time points of producing, handling and storage, processing and packaging, distribution, retail, wholesale, and consumption would contribute to increasing food safety.

## 5. Conclusions

The results of this study suggest that our model can be used to assess the risk level of establishments involved in the food supply chain. A risk level-based classification of laboratories may become a decision-making tool for the competent public health authority regarding food quality management in the context of public and private stakeholders. Indeed, different types (and subtypes) of deviations were identified at the SLs, some of which were considered statistically significant, eliciting different levels of risk. This research denotes the importance of Type 1 deviations that either belong to Subtype a (technic requirements) or b (technic or management requirements), which means that either compromise the reliability of the result and may be questioned by performing the necessary test or calculations incorrectly, or without following the instructions indicated, failures to follow the current technical procedure of the test. This study also highlights the importance of implementing quality management systems to increase quality assurance in control policies and methodologies.

## Figures and Tables

**Table 1:** Description of risk level.

Risk Level	Number of Events (%)	Description
Imminent	≥65	Failure in the process potentially affects the purity, sanitary integrity or accuracy of the final product. Implies non-compliance of legal requirements or a serious impact on health of the consumer. Major corrective actions are required.
High	49–64	The results of the process or product does not meet standard specifications, the results warrant rejection of the product. Major corrective actions are required.
Medium	33–48	The product quality can potentially be found as compromised. Further research is required, corroboration of its quality before releasing the final product. Corrective actions are required.
Low	17–32	It does not affect the quality of the final product, but there are deviations of procedures. Failure at this level may lead to dissatisfaction of the final product. Minor corrective action may be necessary.
Insignificant	≤16	Quality practices assurance and standard procedures are not affected. No further actions are required.

A 5-point risk level scale was used. The number of events indicates the percentage of “practices” detected during audits and encompassed the different scopes of the QMS. Description of risk level was based on pre-established parameters according to Cartín-Rojas et al. [11] with brief modifications of Villegas-Pérez et al. [10].

**Table 2:** Eigenvalues.

	F1	F2	F3	F4
Eigenvalue	14.3722821	11.3529135	9.84697951	0.09532183
Discrimination (%)	40.2951801	31.8298577	27.607711	0.26725124
Cumulative %	40.2951801	72.1250377	99.7327488	100
Bartlett’s statistic	22,917.094	14,806.838	7345.60673	270.231141
*p*-value	<0.001	<0.001	<0.001	<0.001

Eigenvalues and cumulative variability explanatory power of the four discriminant functions revealed by CDA.

**Table 3:** Functions at the centroids for each risk level across the main discriminant functions (F1 to F4).

	F1	F2	F3	F4
Imminent	22.9265	−7.8755	14.9096	0.0023
High	14.5552	4.4517	−13.9543	0.0024
Medium	0.4245	11.6543	4.2327	0.0031
Low	−0.9280	−0.9684	−0.1731	3.0284
Insignificant	−0.8611	−0.8815	−0.1555	−0.0356

## Data Availability

The data that support the findings of this study are available from the corresponding author upon reasonable request.

## Supplementary Materials

The following supporting information can be downloaded at: https://www.mdpi.com/article/10.3390/ani14192802/s1, Table S1: Classification of Deviations by Type and Subtype Based on Findings; Table S2: Unidimensional test of equality of the means of the deviations (Types, subtypes, and their frequencies). Variables marked in red and bold were deemed non-significant (*p* > 0.05).
