# Supplementary material for: Evaluating Procedure-Linked Risk Determinants in Trichinella spp. Inspection under a Quality Management System in Southern Spain
Source: Animals (Basel). 2024 Sep 27;14(19):2802. doi: 10.3390/ani14192802 (PMC11476041; doi:10.3390/ani14192802)
Supplement: Supplementary file 1 [file animals-14-02802-s001.zip › Table S1.pdf]

Table S1. Classification of Deviations by Type and Subtype Based on Findings

Classification of findings affecting QMS requirements	
DEVIATIONS (According to severity of the finding)	Nonconformities (NC)
	Observations (OB))
AMBIT (According to affected requirement)	*Technical Requirements (TR)*
	Management Requirements (MR)
TYPES (Depending on affected component)	SUBTYPES
TYPE 1<br>Affects TECHNIQUE and/or trial information	SUBTYPE (a) The reliability of the result may be questioned by performing the necessary test or calculations incorrectly or without following the instructions indicated. Failure to follow the current Technical Procedure of the test. Use material and/or equipment that is not supported, uncontrolled or deficient.
	SUBTYPE (b) Do not record in the test report data that could only be known if they are collected in the test report (i.e., temperature, sieve weight, digestion %, identification of samples, reagents and/or equipment if more than one is in use). Inappropriately use registry spaces. Untraceable information exists.
TYPE 2<br>Affects EQUIPMENT, MATERIAL, REAGENTS	SUBTYPE (a) There is no Maintenance and Calibration Plan (MANCA Plan)
	SUBTYPE (b) They affect equipment and / or consumables in their identification or registration, technical characteristics, use, operation, control, high or low, without compromising the reliability of the result.
	SUBTYPE (c) Some consumable equipment/material is missing.
	SUBTYPE (d) Equipment instructions are missing or not located/provided.
	SUBTYPE (e) There are equipment/material in poor condition.
	SUBTYPE (f) There is a lack of reagents for testing and/or for the disposal of positive samples with no alternative. The reagents are poorly controlled.
TYPE 3<br>Affects QUALIFICATION	NO SUBTYPES. They compromise the evidence of the qualification or training of the person(s) involved in the performance of the technique and/or the interpretation of the result.
TYPE 4<br>Affects QUALITY ASSURANCE	SUBTYPE (a) They affect internal quality controls (ICC) or external quality controls.
	SUBTYPE (b) They affect the Corrective Action Plan (PAC) and/or the management of deviations.
	SUBTYPE (c) They concern calibrations, verifications, maintenance and/or calibration labels, including the control of information or metrological traceability.
	SUBTYPE (d) They affect the quality certificates of the equipment or technical sheets of the reagents.
	SUBTYPE (e) They affect the ISO certificates of suppliers.
	SUBTYPE (f) They affect the record of deviations.
	SUBTYPE (g) They affect the conservation of reagents.

TYPE 5<br>Affects RECORDS, FORMATS AND OTHER DOCUMENTS	SUBTYPE (a) Unfilled forms. Data is missing, erroneous or illegible. There is a different signature than the one indicated. Data are missing from the test report not included in the TYPE 1 deviation.
	SUBTYPE (b) Missing, not locating/contributing or not using current data record formats (FLS). Do not use the current primary data logging format.
	SUBTYPE (c) Missing, not located/provided or using technical instructions (ITLS) in force.
	SUBTYPE (d) The technical procedure (PTLS) in force and/or the applicable regulations (EU Regulation) are missing, not located or provided, but it does not imply that the test is carried out incorrectly.
	SUBTYPE (e) There are deletions or unvalidated corrections. The space of a section in a format is used abusively. Ellipsis and/or quotation marks are used.
	SUBTYPE (f) No control is maintained of current copies of documents and/or obsolete documents or associated records/documents.
TYPE 6<br>Affects OTHER components	SUBTYPE (a) They affect the facilities (availability of hot / cold water, air conditioning, location, furniture, cleaning or access).
	SUBTYPE (b) Improvement options are evident (e.g.: in the identification of samples, in the management of information, use of better-quality consumables, completion and custody of formats)
	SUBTYPE (c) They affect the elimination of positive samples (inadequate or non-existent containers, lack of reagents or system for it).
	SUBTYPE (d) The location of documents or files is not adequate or unknown. There are only equipment instructions in a language that is not Spanish and is unknown to the user.
